# Fast and efficient molecule delivery into *Euglena gracilis* mediated by cell‐penetrating peptide or dimethyl sulfoxide

**DOI:** 10.1002/2211-5463.13592

**Published:** 2023-03-18

**Authors:** Pingwei Gao, Chengfu Sun

**Affiliations:** ^1^ Scientific Research Center Chengdu Medical College China

**Keywords:** cell‐penetrating peptide, DMSO, *Euglena gracilis*, intracellular delivery, pellicle, Pep‐1

## Abstract

This study describes the development of two methods for delivering exogenous materials into *Euglena gracilis*, a unicellular flagellate organism. We report that the use of Pep‐1, a short cell‐penetrating peptide (CPP), or dimethyl sulfoxide (DMSO) can mediate fast and efficient intracellular delivery of exogenous materials into *E. gracilis*, achieving cellular entry efficiency as high as 70–80%. However, compared with human cells, the penetration of this algal cell with CPP requires a much higher concentration of purified proteins. In addition, upon convenient treatment with DMSO, *E. gracilis* cells can efficiently adsorb exogenous proteins and DNA with 10% DMSO as the optimal concentration for *Euglena* cells. Our results provide more options for the *E. gracilis* transformation ‘toolkit box’ and will facilitate future molecular manipulations of this microalgal organism.

## Graphical abstract

Methods for delivering exogenous molecules into *Euglena gracilis* are still limited. We provide here two simple methods with fast and efficient delivery performance for this alga. One method utilized the membrane entry ability of CPP and the other the membrane permeability of DMSO. Both can achieve over 70% delivery efficiency. Our methods promise to be beneficial for *E. gracilis* research.

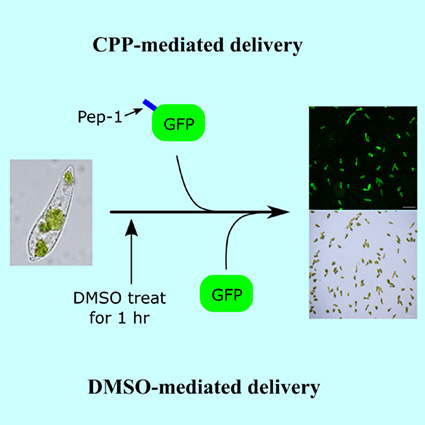

**Abbreviations**
CPP: cell‐penetrating peptide
DAPI: 4′,6‐diamidino‐2‐phenylindole
DMSO: dimethyl sulfoxide
EGFP: enhanced green fluorescent protein
IPTG: isopropyl‐β‐d‐thiogalactoside
kb: kilobase
PBS: phosphate‐buffered saline
PCR: polymerase chain reaction
SDS/PAGE: sodium dodecyl sulfate‐polyacrylamide gel electrophoresis

*Euglena gracilis* is a unicellular photosynthetic flagellate that belongs to the Excavata supergroup of eukaryotes. Unlike its parasitic trypanosomatid cousins of the same supergroup, *E. gracilis* is free‐living and has a three‐layered membranous organization in its chloroplast [1]. As a model organism, *E. gracilis* has been extensively studied on cell motility such as phototaxis and gravitaxis, RNA splicing including chloroplastic group II and III introns, as well as nuclear spliceosomal introns, and other biological processes [2, 3, 4]. In addition, *E. gracilis* is an important bioreactor for biomass production due to its nutrient value [2].

To date, delivery of exogenous materials into *E. gracilis* cells can be realized with four methods: *Agrobacterium*‐mediated nuclear transformation, electroporation, biolistic bombardment, and single‐cell microinjection [5]. Although these methods are feasible with appreciable efficiency for *E. gracilis* transformation, they require sophisticated apparatuses with optimized parameters or take a relatively long time to screen positive clones.

Cell‐penetrating peptides (CPPs) are a group of short peptides with cationic or amphipathic residues in composition and can be taken up by cells mainly through translocation and endocytosis [6]. In addition, CPPs confer conjugated exogenous materials, via either covalent or noncovalent interactions, the ability to enter many types of cells. Similar to CPPs, DMSO can affect membrane permeability and is used for membrane‐related activities, such as yeast transformation, and improve membrane penetration efficiency of TAT‐treated cells [7, 8, 9]. Here we described two delivery methods based on CPP and DMSO utilizing their ability to penetrate or disturb cell membranes. Our results suggested that both methods applied to *E. gracilis* cells and achieved fast and efficient delivery results.

## Materials and methods

### Chemicals and microorganisms

*Euglena gracilis* strain FACHB‐848 was ordered from the Freshwater Algae Culture Collection at the Institute of Hydrobiology (Wuhan, China) and cultured according to its instructions in the HUT medium [10]. DMSO was ordered from MP Biomedicals and trypan blue from TargetMol.

### PCR

PCR reactions were conducted with Phanta Super‐Fidelity DNA Polymerase (Vazyme, Nanjing, China). PCR primers used in this work, as listed in Table 1, were synthesized from Tsingke. To check the presence of pPZP211 plasmid in DMSO‐treated *E. gracilis* cells, DNA was extracted from alkaline lysed cells and used for PCR with primers 211‐L5sc_F and mGFP6seq_R. For amplifying the Prp8 fragment, primers (egP8_F/Cy5‐egP8_F/FITC‐egP8_F and egP8_R) were designed according to the *E. gracilis* Prp8 nucleotide sequence (Genbank accession number OP185589) that was previously assembled. After extracting total RNAs from *E. gracilis* cells with Trizol, reverse transcription was performed with FastKing RT Kit With gDNase (TIANGEN, Beijing, China). To visualize the PCR fragment in the cell, the forward primers (Cy5‐egP8_F/FITC‐egP8_F) were additionally labeled with a Cy5 or FITC dye at their 5′ termini synthesized also by Tsingke (Beijing, China).

**Table 1:** Primers used in this research.

Primer	Sequence	Comment
21G‐pep‐1sc_F	AGGAGATATACATATGAAAGAAACCTGGTGGG	For pET21‐PGH
21G‐pep‐1sc_R	ccttgctcacggatccCACTTTACGTTTTTTTTTC	
GP_oF	cggcatggacgagctgtacaagggatccAAAGAAACCTGGTGGGAAACC	For pET21‐GPH
P21sc_R	GGTGGTGGTGCTCGAGCACTTTACGTTTTTTTTTCGGC	
21HGPsc_F	AATGGGTCGCGGATCCCACCACCACCACCACCACGTGAGCAAGGGCGAGGA	For pET21‐HGP
21HGPsc_R	GGTGGTGGTGCTCGAGCTACACTTTACGTTTTTTTTTCGGCTG	
21HPGsc_F	AGGAGATATACATATGCACCACCACCACCACCACAAAGAAACCTGGTGGGAAACC	For pET21‐HPG
21HPGsc_R	GGTGGTGGTGCTCGAGctAgtacagctc	
egP8_F	CAACAACCTGACGGACATCTG	For DMSO transduction
Cy5‐egP8_F	Cy5‐CAACAACCTGACGGACATCTG	
egP8_R	GCTTCTTGTACTTCATGCTCTCC	
211‐L5sc_F	atcctctagagtcgacagggaaacgtctcttttggc	For DMSO transduction
mGFP6seq_R	gggttggccatggaacagg	

### Plasmid construction

**Fig. 1:**
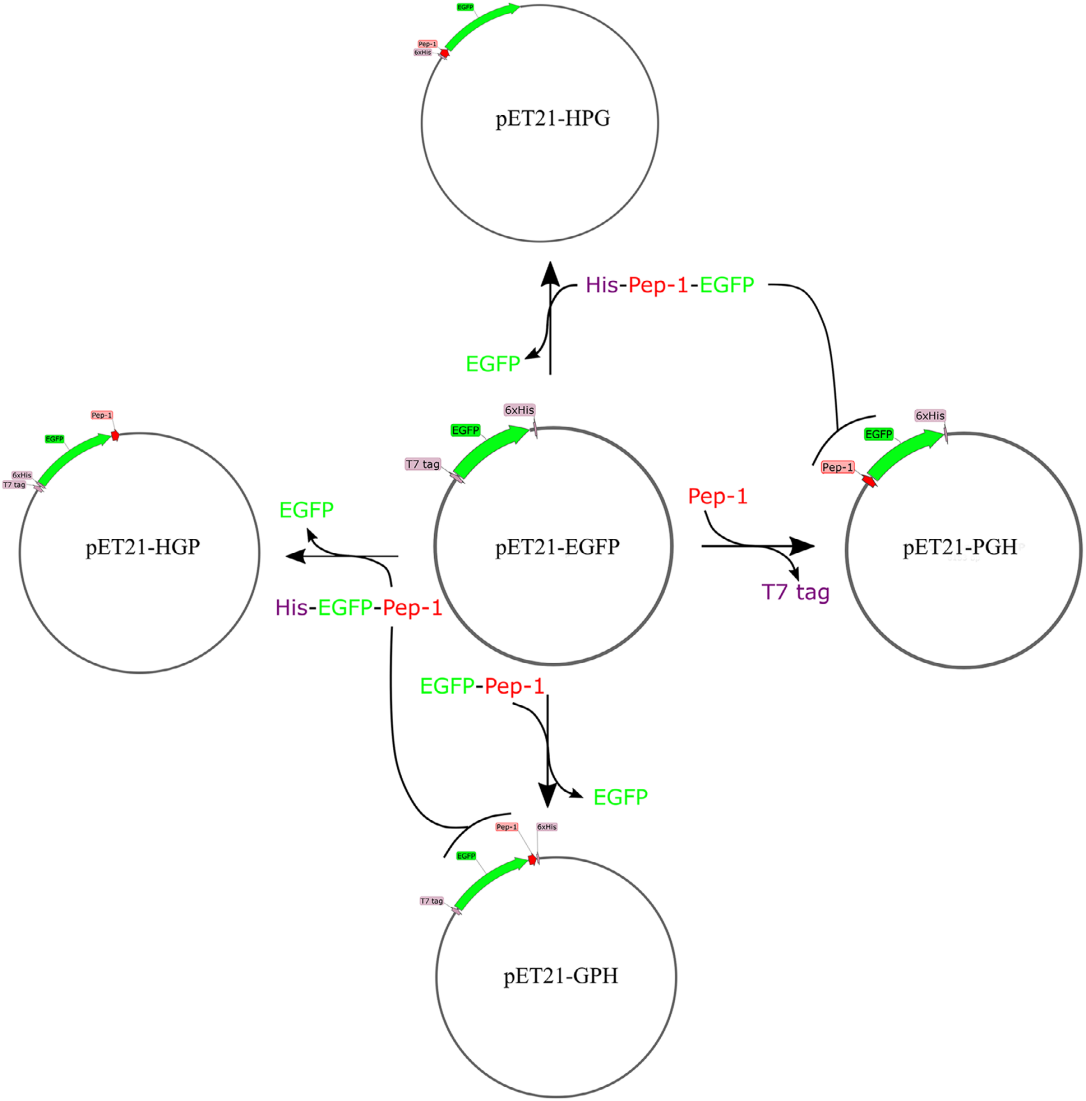
Schematic diagram of the procedure for plasmid construction.

Plasmids used here were constructed with a ligation‐independent cloning method (ClonExpress II One Step Cloning Kit, Vazyme) and based on the pET21‐EGFP plasmid, which contains an EGFP coding sequence flanking with an upstream T7 tag and downstream 6× His residues (Fig. 1). pET21‐PGH was constructed by replacing the T7 tag sequence (MASMTGGQQMG) on pET21‐EGFP with Pep‐1 (LETWWETWWTEWSQPKKKRKV), gene synthesized from Tsingke Biotechnology and amplified with primers (21G‐pep‐1sc_F and 21G‐pep‐1sc_R) except the first Methionine [11, 12]. pET21‐GPH was constructed by inserting the Pep‐1 fragment (amplified with GP_oF and P21sc_R) into pET21‐EGFP. For constructing pET21‐HGP, the GFP‐Pep‐1 fragment was amplified from pET21‐GPH with the forward primer 21HGPsc_F containing the 6× His sequence and the reverse primer 21HGPsc_R containing a stop codon after the Pep‐1 sequence. Subsequently, the amplified GFP‐Pep‐1 fragment was inserted into the BamHI/XhoI‐linearized pET21‐EGFP to generate pET21‐HGP. Similar to pET21‐HGP, pET21a‐HPG was constructed by amplifying the Pep‐1‐GFP fragment from pET21‐PGH with the forward primer 21HPGsc_F containing the 6× His sequence and the reverse primer 21HPGsc_R containing a stop codon after the EGFP sequence, and then inserted into the NdeI/XhoI‐linearized pET21‐EGFP.

### Protein purification and SDS/PAGE

Plasmids were transformed into BL21 (DE3) and expressed upon adding IPTG for 3 h at 37 °C. Subsequently, cells were harvested by centrifugation, dissolved in buffer A (20 mm Tris–HCl, pH 8.0, and 25 mm NaCl) supplemented with 1 mm phenylmethylsulfonyl fluoride (PMSF) and then broken by sonification. Soluble His‐tagged Pep‐1 fusion proteins were affinity purified with Ni‐NTA resin. Thirty millimolar imidazole was used to remove unbound proteins from the resin, and 300 mm to elute Pep‐1 proteins. The eluted proteins were further concentrated by ultrafiltration and dialyzed against phosphate buffer saline (PBS). Purified proteins were applied to 10% SDS/PAGE and visualized with coomassie staining. For the delivery experiment, proteins were diluted to different working concentrations with an HUT medium.

### Intracellular delivery experiment

*Euglena gracilis* cells were collected by centrifuging at 1000 ***g*** for 1 min and washed once with flesh HUT medium. Subsequently, cells were added with prepared proteins of different concentrations to a final cell density of 1 O.D (0.86 × 10^6^ cells·mL^−1^). Cells were then incubated at 37 °C (for CPP) or 25 °C (for DMSO) at different times. For DMSO treatment, cells were incubated with an HUT medium containing different concentrations of DMSO for 1 h, then washed with PBS three times before adding exogenous proteins or nucleic acids.

### Cell viability assay

After treatments of electroporation, PGH protein or DMSO, cells were stained with 4 mm trypan blue for 3 min, centrifuged for 1 min at 1000 ***g***, washed with PBS, and then observed under a microscope.

### Microscopy

*Euglena gracilis* cells were collected, washed with PBS several times, and observed under the fluorescence microscope BX63 (Olympus) at 20× or 100× magnification. For DAPI staining, cells were fixed with 4% paraformaldehyde for 20 min, treated with 0.5% Triton X‐100 for 15 min, and stained with 1 μg·mL^−1^ DAPI for 5 min before observation.

### Statistical analyses

Experiments for both Pep‐1 and DMSO delivery were repeated at least three times and statistical analyses were performed with prism7 (GraphPad, Boston, MA, USA). Using a significance level set at 95%. Based on statistically significant differences, the Tukey's multiple post‐test (for PGH delivery) and the one‐way analysis of variance test (for DMSO delivery) were used to analyze the data of each group. For PGH and DMSO delivery, the difference was considered statistically significant when *P* < 0.0001 and *P* < 0.05, respectively.

## Results

### Purification of Pep1 fusion GFP proteins

**Fig. 2:**
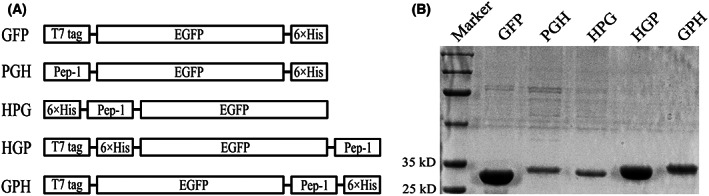
Purified proteins used in this research. (A) Schematic representation of the Pep‐1 fusion protein structures and the control GFP protein expressed from the pET21‐EGFP plasmid. (B) 10% SDS/PAGE analysis of purified proteins.

To investigate CPPs‐mediated delivery of exogenous materials into *E. gracilis*, the 21‐amino acids amphipathic CPP peptide Pep‐1 was selected for our research [11]. The nucleic acids sequence of Pep‐1 was synthesized according to Choi *et al*. [12]. Plasmid pET21‐EGFP, which contained an EGFP coding sequence, was used as a template for constructing Pep‐1‐related plasmids as mentioned in Materials and methods (Fig. 1). Pep‐1 was inserted into pET21‐EGFP between the T7 tag and EGFP to obtain the plasmid pET21‐PGH (Figs 1 and 2A). Subsequently, this plasmid was transformed in BL21(DE3) strain for expression of the 31‐kD PGH protein and purification via Ni‐NTA affinity chromatography (Fig. 2B).

Besides the pET21‐PGH plasmid, three other Pep‐1‐related plasmids were constructed for the purpose mentioned below. These three plasmids, pET21‐HPG, pET21‐HGP, and pET21‐PGH, differed only in the positions of His tag and Pep‐1 (Figs 1 and 2A). Under the same experimental conditions as for pET21‐PGH, these plasmids were successfully expressed, and the corresponding proteins with similar sizes were expressed and purified (Fig. 2B). Moreover, a pET21‐GPH plasmid without the T7 tag sequence was constructed. However, we did not detect the protein expression of this plasmid after IPTG induction, suggesting that the T7 tag sequence is required for GPH expression, agreeing with a previous report [13].

### Intracellular delivery of PGH into *E. gracilis* cells

**Fig. 3:**
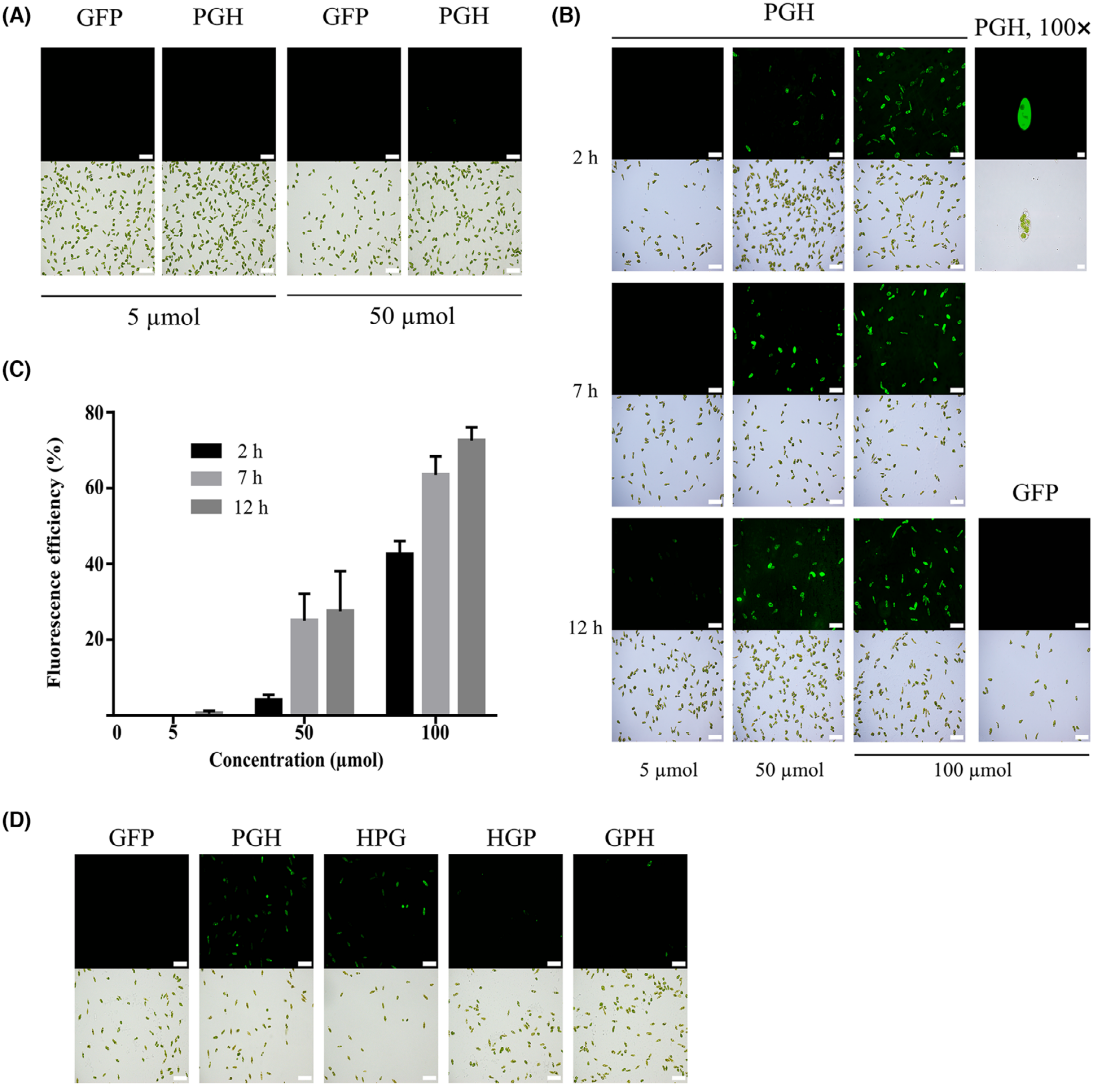
Intracellular delivery of the Pep‐1 fusion protein PGH into *E. gracilis* cells. (A) Intracellular delivery of PGH at the physiological temperature. Five or 50 μm PGH protein was added to *E. gracilis* cells and incubated at 25 °C for 12 h. (B) Time‐ and concentration‐dependent intracellular delivery of PGH into *E. gracilis* cells. Cells were treated with different concentrations of PGH protein as indicated for 2, 7, or 12 h at 37 °C before observing under a microscope. Micrographs were taken at 20× magnification except an enlarged one indicated with 100× magnification on the right side of the panel. (C) Quantification of PGH delivery efficiency in *E. gracilis* cells. Fluorescence efficiency is defined as the ratio of cells with fluorescence to total cells. Error bars are the SD of three independent experiments. (D) Intracellular delivery of four Pep‐1 fusion proteins with Pep‐1 tag located at either the N‐ or C‐terminus of the respective protein. For (A), (B), and (D), results of the Pep‐1‐free GFP protein, used as a control, are shown along with those of Pep‐1 fusion proteins. For each set of images, the fluorescent view is on the top and the bright field view is on the bottom. Scale bar is 10 μm for the 100× magnification of PGH in (B) and 50 μm for all other images.

Pep‐1 fusion protein could enter cells as low as 0.25 μm and as short as 15 min [12]. However, when 5 μm of PGH was added to *E. gracilis* cells, no fluorescence signal was observed after incubation at 25 °C for 12 h. Only after increasing the concentration of PGH to 50 μm could we observe very few fluorescence signals (Fig. 3A). Because human cells are usually cultured at 37 °C, we speculated PGH delivery might be enhanced in *E. gracilis* cells at this higher temperature. Indeed, much more fluorescence signals were found at 37 °C than at 25 °C (compare Fig. 3B to Fig. 3A). Accordingly, we conducted Pep‐1 fusion protein delivery at this higher temperature hereinafter.

Three concentrations of PGH for *E. gracilis* delivery were tested (Fig. 3B). When 5 μm of PGH was added to *E. gracilis* cells, no fluorescence signal was observed after 2 and 7 h of incubation. Even after 12 h of incubation, only very few cells (~ 1%) exhibit the fluorescence signals (Fig. 3B,C). Consequently, the PGH concentration was added to 50 and 100 μm. Our data indicated more than 25% and 60% delivery efficiencies at 50 and 100 μm of PGH after 7 h of incubation, respectively (Fig. 3B,C). Longer incubation up to 12 h only slightly increased the delivery efficiency (70% for 100 μm). We noticed that the transduced PGH proteins were evenly distributed in the cell except some dark areas overlapped with chloroplasts (100× magnification image in Fig. 3B). Although the PGH concentration added to *E. gracilis* cells was much higher than that to human cells, our results showed that PGH‐transduced *E. gracilis* cells remained motile but less active than they were at physiological temperature. The viability of PGH‐transduced cells was also confirmed by staining with trypan blue (Fig. 5). Compared with electroporated cells, only a few PGH‐treated cells (~ 5%) were stained blue. For all these delivery conditions, our results detected no fluorescence signal with the Pep‐1‐free GFP protein (Fig. 3B).

### Position of Pep‐1 influenced delivery efficiency

Besides the above PGH protein, three other Pep‐1‐related plasmids were constructed and respective proteins were purified (Fig. 2). As mentioned above, these proteins only differ in Pep‐1 and His tag positions. The purpose of the purification of these proteins was to investigate the influence of Pep‐1 position on delivery efficiency. Indeed, upon adding these three proteins, together with the PGH mentioned above, to *E. gracilis* cells, our findings suggested that delivery efficiencies of PGH and HPG were much higher than those of HGP and GPH (Fig. 3D). This result suggested that N‐terminal Pep‐1 promoted intracellular delivery more efficiently, consistent with a previous report [14].

### Intracellular delivery mediated by DMSO

**Fig. 4:**
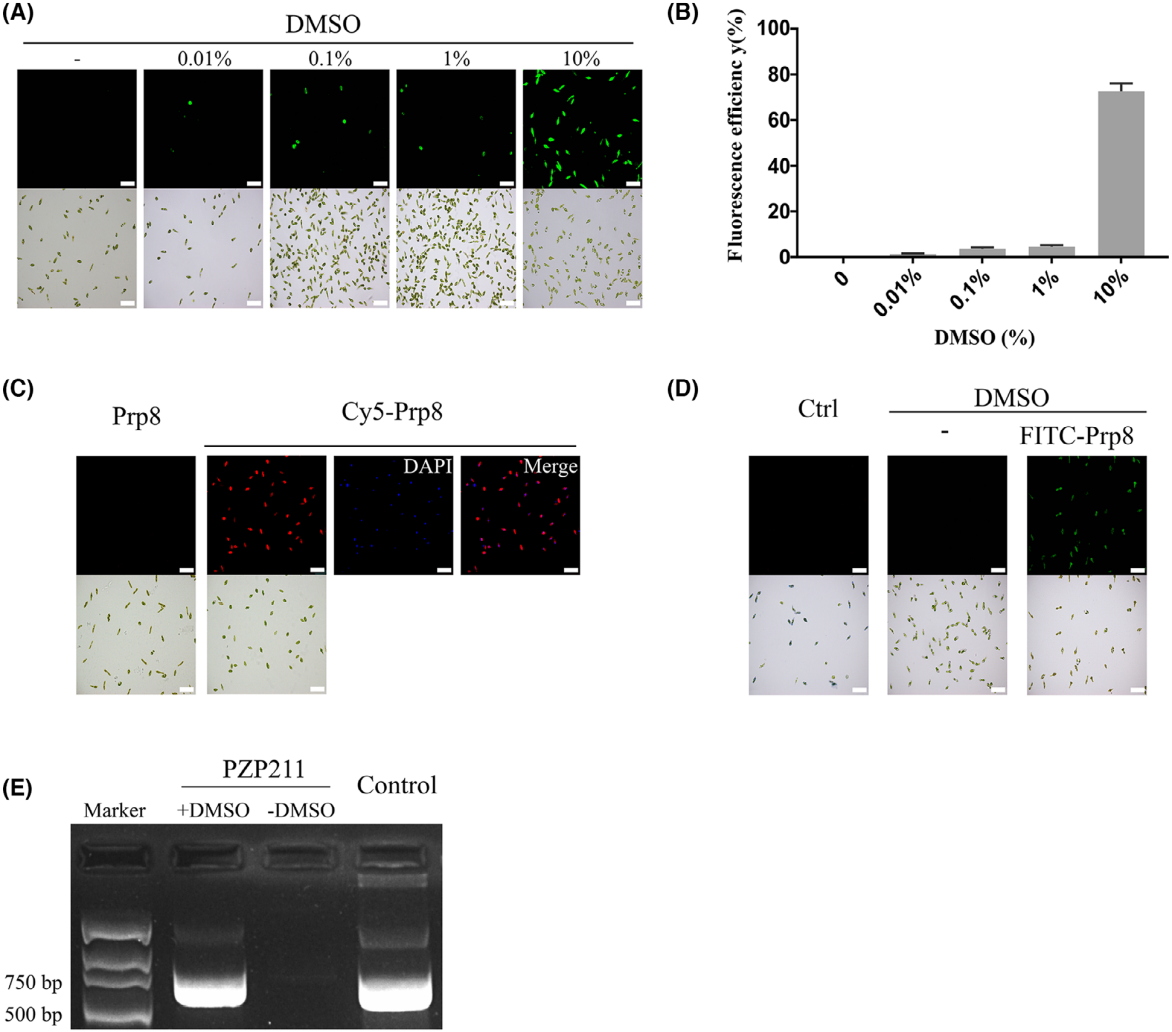
Intracellular delivery of exogenous proteins and DNAs into *E. gracilis* cells with DMSO. (A) Transduction efficiency of Pep‐1 tag‐free GFP in *E. gracilis* cells treated with different concentrations of DMSO. (B) Quantification of GFP delivery efficiency. Error bars are the SD of three independent experiments. (C) Intracellular delivery of a DNA fragment. The 1.7‐kb Prp8 fragment with or without a Cy5 label was added at a final concentration of 40 ng·μL^−1^ to the *E. gracilis* cells after 10% DMSO treatment. For cell delivery with Cy5‐Prp8, locations of nuclei stained with DAPI were also shown and superimposed with the Cy5 fluorescent signals. (D) Trypan blue staining of DMSO‐treated cells with FITC‐labeled Prp8 fragment. (E) Examination of intracellular delivery of a pPZP211 plasmid using PCR. A band of 736 bp in size was recovered only from cells treated with 10% DMSO. PCR from the plasmid served as a positive control. For each set of images in (A) and (C), the fluorescent view is on the top and the bright field view is on the bottom. Scale bar: 50 μm.

**Fig. 5:**
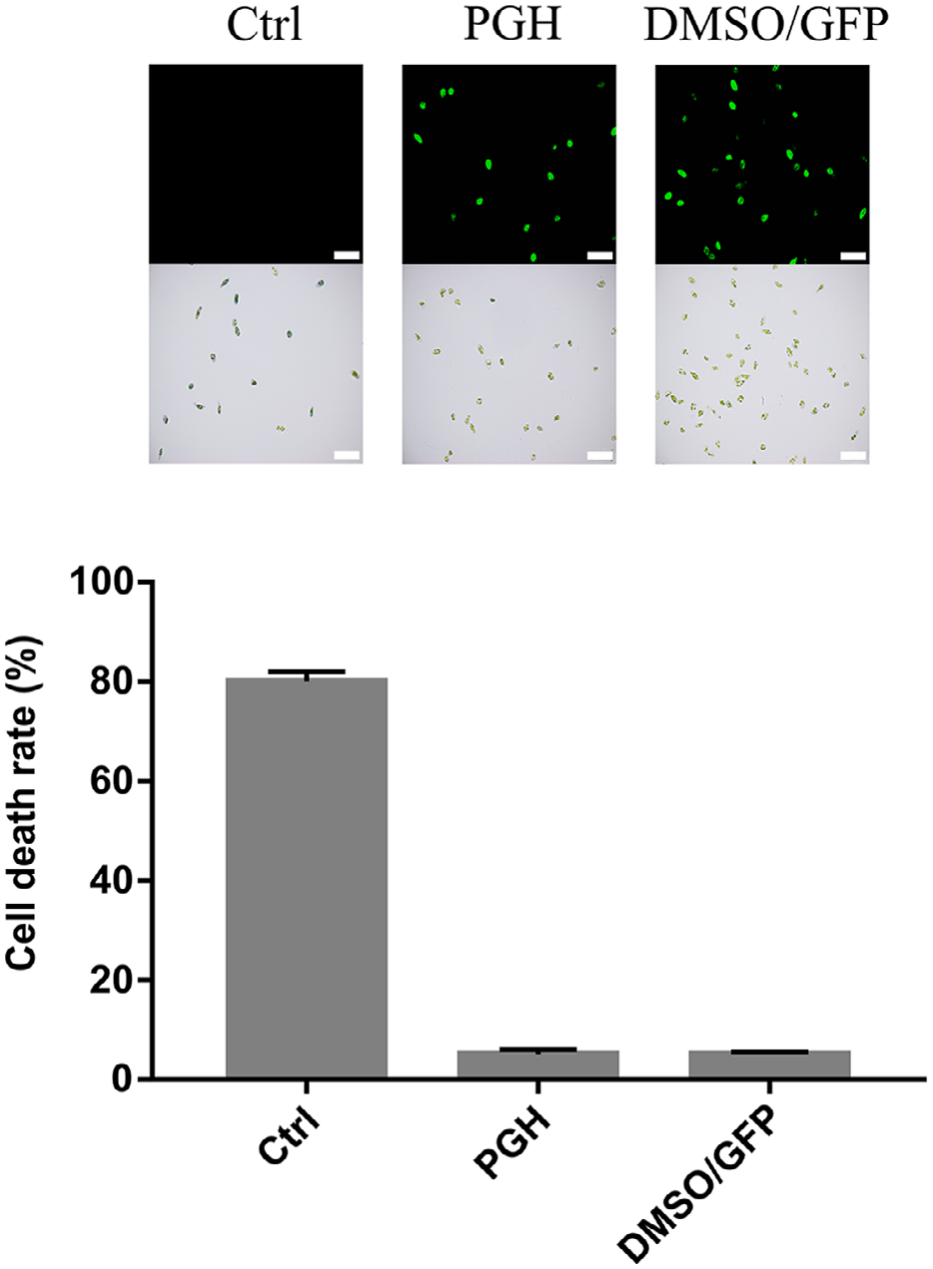
Viability of *E. gracilis* cells after treatment with PGH or DMSO/GFP. Cells were stained with trypan blue, and the dead cells were stained blue. Electroporated *E. gracilis* cells were used as a positive control. Error bars are the SD of three independent experiments. Scale bar: 50 μm.

As mentioned above, a higher temperature (37 °C vs. 25 °C) promoted the delivery of PGH. Because higher temperatures increase the fluidity of cellular membranes, we speculated reagents that affect membrane fluidity might also influence the delivery of exogenous materials. Here, this idea was tested with DMSO, a commonly used laboratory reagent that can enhance membrane permeability. *E. gracilis* cells were treated with different concentrations of DMSO for 1 h. After removing DMSO by washing with PBS, cells were added with the GFP protein without the Pep‐1 tag and incubated for 12 h. The permeation of GFP in *E. gracilis* cells also exhibited a concentration‐dependent manner of DMSO, indicative of a similar effect as Pep‐1 (Fig. 4A,B). With 10% DMSO, ~ 80% delivery efficiency was observed with high viability, as confirmed by trypan blue staining (Fig. 5).

Besides the above delivery with proteins, DNAs were also examined. A pair of primers (Table 1) was designed to amplify a 1.7‐kb fragment of the *E. gracilis* Prp8. To directly observe the intracellular localization of this DNA fragment just as that of the above fluorescent protein, a fluorescent dye Cy5 was conjugated with the 5′ of the forward primer. With the same delivery procedure as GFP, the red fluorescent signals of this DNA fragment in *E. gracilis* cells were also observed under the microscope (Fig. 4C). Notably, the intracellular delivery efficiency of this DNA fragment was even higher than that of the above GFP protein. Additionally, to quench any fluorescent signals attached to the cell surface, cells were stained with trypan blue after being treated with DMSO and FITC‐labeled. Our findings revealed that the cellular localization of the Prp8 fragment displayed the same patterns as above (Fig. 4D).

Finally, to examine the feasibility of plasmid delivery into *E. gracilis* cells with DMSO, a plasmid of 12 kb in size bearing the pPZP211 backbone was used for this purpose. Upon DMSO treatment for 1 h and removal by washing with PBS, the plasmid was added to *E. gracilis* cells at a final concentration of 10 ng·μL^−1^. After 3 days of culture, cells were washed, and cellular DNAs were extracted. A 736‐bp fragment was detected from DMSO‐treated cells (Fig. 4E). In comparison, no DNA was amplified from cells without DMSO treatment. This result suggested that a plasmid as large as 12 kb can enter *E. gracilis* cells with the help of DMSO.

## Discussion

As an evolutionarily unique and model organism, *E. gracilis* has been studied for, from scientific questions to biotechnology solutions. All these studies on *E. gracilis* demand more methodologies. We here developed CPP‐ and DMSO‐mediated transduction methods for delivering exogenous materials into this algal cell. Compared with available methods, such as Agrobacterium‐mediated nuclear transformation, electroporation, and microinjection, the methods described here are fast, convenient, and efficient.

Under our experimental conditions, the delivery of Pep‐1 conjugated PGH to *E. gracilis* cells resulted in approximately 70% efficiency. Notably, *E. gracilis* requires a much high concentration for cell entry than human cells. Besides Pep‐1, an arginine‐rich cationic CPP peptide TAT (GRKKRRQRRR) was tested, and the results indicated delivery concentration and efficiency of TAT‐EGFP are comparable to PGH. A unique microtubular structure underneath the cell membrane in *E. gracilis* cells is termed the pellicle [15]. We speculated the pellicle might hinder the entry of CPP, and only a higher concentration of CPPs can break through this barrier. It will be interesting to check whether the CPP delivery efficiency can be increased to that of humans after disrupting the pellicle. Because the molecular composition of the pellicle is so far unclear, this hypothesis cannot be examined here [16]. Although it appears *E. gracilis* cells are refractory to CPP delivery, further investigation and optimization will make this delivery peptide more applicable.

For DMSO delivery, ~ 80% of *E. gracilis* cells displayed the fluorescent signals of exogenous GFP protein and Cy5‐labeled DNA fragment with 10% DMSO. Also, higher concentrations of DMSO were tested. Although these treated cells showed a relatively higher delivery efficiency, they were more inactive than 10% DMSO‐treated cells. In addition, a previous study in human cells found that DMSO aided the penetration of GFP with a CPP peptide but failed to do so without the peptide [9]. By contrast, our study showed that DMSO is effective for *E. gracilis* cell delivery even without CPP. Therefore, DMSO is applicable as an independent delivery method in *E. gracilis* cells, similar to its role in yeast transformation [8].

Usually, delivery of RNA or large ribonucleoprotein complexes in *E. gracilis* depends on traditional electroporation [17, 18]. Although our methods tested only protein and DNA molecules here in *E. gracilis*, they should be suitable for delivering other exogenous materials. In addition, our methods may be applied to other algals as well.

## Conflict of interest

The authors declare no conflict of interest.

## Author contributions

PG conducts all experiments, analyzes the data and writes the original manuscript draft. CS conceptualizes and supervises the project, analyzes the data, writes and edits the manuscript. All authors have read and approved the submitted manuscript.

## Data Availability

The supporting data are available from the corresponding author upon request.
